# Galangin Suppresses Renal Inflammation via the Inhibition of NF-*κ*B, PI3K/AKT and NLRP3 in Uric Acid Treated NRK-52E Tubular Epithelial Cells

**DOI:** 10.1155/2019/3018357

**Published:** 2019-05-26

**Authors:** Hao Lu, Hui Yao, Rong Zou, Xiwen Chen, Hanlin Xu

**Affiliations:** School of Pharmaceuticals, Hubei University of Chinese Medicine, No. 1 HuangJiaHu Road West, Hongshan District, Wuhan 430065, China

## Abstract

Renal inflammation can result in renal injury. Uric acid (UA) is the final product of purine metabolism in humans and because of the lack of urate oxidase, UA may accumulate in tissues, including kidney, causing inflammation. Galangin was isolated from a traditional Chinese medicine plant and possesses several beneficial effects, working as an anti-oxidant, anti-mutagenic, anti-tumor, anti-inflammatory, anti-microbial, and anti-viral agent. Therefore, this study aimed at investigating the molecular mechanism of galangin in the attenuation of UA induced renal inflammation in normal rat kidney epithelial cells NRK-52E. Our findings suggested that galangin treatment efficiently protected NRK-52E cells against UA induced renal inflammation by decreasing tumor necrosis factor (TNF)-*α*, interleukin (IL)-1*β*, IL-18, prostaglandin E2 (PGE2), and nitric oxide (NO) release, and it inhibited nitric oxide synthase (iNOS), prostaglandin endoperoxide synthase 2 (PTGS2), TNF-*α*, IL-1*β*, and IL-18 mRNA expression. In addition, galangin was not exerting any cytotoxicity at the concentrations that were effective against inflammation as assessed by CCK8 assay. Moreover, western blotting showed that galangin treatment effectively inhibited nuclear factor-kappa B (NF-*κ*B), phosphatidylinositol 3 kinase (PI3K)/protein kinase B (AKT) and nucleotide-binding domain- (NOD-) like receptor protein 3 (NLRP3) signaling pathway activation. Taken together, these findings suggested that galangin plays a pivotal role in renal inflammation by suppressing inflammatory responses, which might be closely associated with the inhibition of NLRP3 inflammasome, NF-*κ*B and PI3K/AKT signaling pathway activation.

## 1. Introduction

Renal inflammation, which is characterized by the infiltration of inflammatory cells into the renal parenchyma, is an imperative pathologic process in the evolution of renal injury [1]. A growing number of epidemiologic and experimental evidences suggest that several endogenous substances, especially uric acid (UA), the final product of purine metabolism in humans and higher primates[2], play an important role in mediating inflammation stimulated by a pathologic insult and tissue damage. Because of the lack of urate oxidase, also called uricase, in humans, UA may accumulate in tissues [3]. Deposited UA crystal causes renal inflammation by depositing intraluminal crystals in the collecting duct of the kidney [4, 5]. Normal rat kidney epithelial cells (NRK-52E) modulate both the innate and adaptive immune response and exert a pro-inflammatory effect with the release of several cytokines under specific stimuli [6]. Exposure of NRK-52E cells to UA induces inflammation that is characterized by the overproduction of pro-inflammatory cytokines and mediators. Increasing evidence shows that various pro-inflammatory cytokines and mediators, including TNF-*α*, IL-1*β*, IL-18, NO, and PGE2, are involved in UA induced renal inflammation, chronic kidney disease, and even renal failure [7–9]. Accordingly, the suppression of the inflammatory response might be a potential prevention of the development of renal inflammation.

Many previous reports showed that multiple signaling pathways, including NF-*κ*B, PI3K/AKT, and NLRP3 inflammasome, play an essential role in the regulation of inflammatory responses. On one hand, NF-*κ*B, a crucial transcription factor in the regulation of many inflammatory mediators, is essential for host defense and also mediates these pro-inflammatory cytokines and mediators expression [10–13]. Moreover, PI3K/Akt is also closely related to inflammation [14–16]. Activation of PI3K and its downstream target Akt is essential for NF-*κ*B activation [17]. On the other hand, NLRP3, along with Apoptosis-associated speck-like protein containing a caspase recruitment domain (ASC) and Cysteinyl aspartate-specific proteinase-1 (caspase-1), forms a multiprotein complex called the NLRP3 inflammasome. The NLRP3 inflammasome can activate caspase-1 in response to a number of diverse stimuli including UA and bacterial pore forming toxins, resulting in the secretion of the pro-inflammatory cytokines/mediators. Hence, the inhibition of NF-*κ*B, PI3K/AKT signaling pathway and NLRP3 inflammasome activation, might contribute to reduce renal inflammation.

Galangin (3,5,7-trihydroxyflavone), a member of the flavonol class of flavonoids, was isolated from the traditional Chinese medicine plant* Alpinia officinarum* Hance, a plant which has been used as both spice and herbal medicine for a variety of ailments in China [18]. Previous studies demonstrated significant beneficial effects of galangin, working as an anti-oxidant, anti-mutagenic, anti-tumor, anti-inflammatory, anti-microbial, and anti-viral agent in vitro and in vivo[19–22]. More specifically, the anti-inflammatory effect of galangin was reported in arthritis, asthma, paw edema, acute lung injury, and acute kidney injury. Moreover, galangin suppresses renal inflammation in mice by inhibiting NF-*κ*B activation [23]. In the present study, we investigated whether galangin could alleviate UA induced NRK-52E cell inflammation through the inhibition of NF-*κ*B, PI3K/AKT signaling pathway and NLRP3 inflammasome activation, and the molecular mechanisms were also clarified.

## 2. Materials and Methods

### 2.1. Cell Culture and Treatment

NRK-52E cells were purchased from the China Center for Type Culture Collection (CCTC, Shanghai, China). They were routinely cultured in Dulbecco's modified Eagle's medium (DMEM) (Gibco, Gaithersburg, MD, USA) supplemented with 10% fetal bovine serum (Gibco), 100 *μ*g/ml streptomycin, and 100 U/ml penicillin. Cells were incubated at 37°C in a humidified 5% CO_2_ atmosphere. Galangin (aladdin, Shanghai, China) was dissolved in DMEM as a stock solution of 100 mg/ml and stored at 4°C in the dark until use. UA (aladdin, Shanghai, China) was dissolved in phosphate buffered saline (PBS) (Thermo Fisher Scientific, Waltham, MA, USA) as a stock solution of 5 mg/ml and stored at 4°C in the dark until use. NRK-52E cells were treated with galangin at different concentrations (5, 10, 20, or 40 *μ*g/ml) in the presence or absence of UA (50 *μ*g/ml) for 3, 12, or 24 h. Untreated cells were used as control.

### 2.2. Cell Viability

NRE-52E cells were seeded into a 96-well plate at approximately 1×10^4^ cells per well in 100 *μ*l culture medium per well and treated with galangin at different concentrations for 24 h with or without UA as explained in the previous paragraph. Then, their viability was determined by Cell Counting Kit-8 (CCK-8) (Engreen Biosystem Co. Ltd, Beijing, China). Briefly, at the end of the incubation time 10 *μ*l of CCK-8 solution was added into each well and incubated at 37°C for 4 h. Absorbance in each well was measured in a Model 680 microplate reader (Bio-Rad, Hercules, CA, USA) at 450 nm.

### 2.3. Measurement of Pro-Inflammatory Mediators' Release

NRK-52E cells (5×10^5^ cells/ml) were seeded into 12-well plates in 2 ml culture medium per well and routinely cultured until surface adherence was achieved. Cells were treated with UA (50 *μ*g/ml) in the presence of galangin (5, 10, or 20 *μ*g/ml) for 12 h and untreated cells were considered as the control. The culture medium from the control and treated cells was collected and centrifuged at 1000 rpm/min for 20 min at 4°C, and the obtained cell-free supernatant was stored at -80°C until further use. TNF-*α*, IL-1*β*, PGE2, and IL-18 levels were measured in the cell-free supernatant using ELISA (Elabscience Biotechnology Co. Ltd, Wuhan, China) according to the manufacturer's instructions and absorbance was read at 450 nm. NO level was measured by Griess reagent (Beyotime Institute of Biotechnology, Haimen, China) according to the manufacturer's instructions and absorbance was read at 570 nm.

### 2.4. RT-qPCR

NRK-52E cells were seeded as described above and treated with UA (50 *μ*g/ml) in the presence of galangin (5, 10, or 20 *μ*g/ml) for 12 h and untreated cells were considered as the control. Total RNA was extracted by Trizol RNA isolation reagent (TIANGEN Biotech, Beijing China) and its purity was assessed by the ratio at 260 and 280 nm. Total RNA was reverse-transcribed using the RevertAid FirstStrand cDNA Synthesis Kit (Toyobo, Osaka, Japan). cDNA was used as a PCR template for RT-qPCR amplification using the KAPA SYBR FAST qPCR Kit (Kapa Biosystems, Wilmington, MA, USA) in a BIO-RAD CFX96 touch q-PCR system (Bio-Rad). *β*-actin was used as a reference gene and PCR primer sequences, designed by the software primer premier 3.0, and synthesized by TsingKe, Beijing, were as follows (forward primer and reverse primer, respectively): *β*-actin: 5′-AACCTTCTTGCAGCTCCTCC-3′, 5′-TACCCACCATCACACCCTGG′; INOS2: 5′-TGGTGAGGGGACTGGACTTT-3′, 5′-TGTTGGGCTGGGAATAGCAC-3′; PTGS2: 5′-CTCAGCCATGCAGCAAATCC-3′, 5′-GGGTGGGCTTCAGCAGTAAT-3′; TNF-*α*: 5′-ATGGGCTCCCTCTCATCAGT-3′, 5′-GCTTGGTGGTTTGCTACGAC-3′; IL-1*β*: 5′-CTTTGAAGAAGAGCCCGTCC-3′, 5′-CCAAGGCCACAGGGATTTTG-3′; NLRP3: 5′-GACCAGCCAGAGTGGAATGATG-3′, 5′-CTGTTGAGGTCCACGCTCTC-3′; IL-18: 5′-GCCATGTCAGAAGAAGGCTCT-3′, 5′-GGATTCGTTGGCTGTTCGGT-3′. RT-qPCR amplification was performed with initial denaturation at 95°C for 3 min and 40 cycles at 95°C for 10 s, 60°C for 30 s, and 72°C at 30 s. Relative mRNA expression was calculated using the 2^−ΔΔCt^ method.

### 2.5. Western Blotting

NRK-52E cells (1×10^6^ cells/ml) were seeded into 6-well plates in 5 ml culture medium per well and treated with UA (50 *μ*g/ml) in the presence of galangin (5, 10, or 20 *μ*g/ml) for 12 h and untreated cells were considered as the control. Cells were washed twice with cold PBS before lysis, sonicated, and homogenized in radioimmunoprecipitation assay (RIPA) buffer (Beyotime) containing 1 mM phenylmethanesulfonyl fluoride (PMSF) (Beyotime). The protein concentration was determined using the bicinchoninic acid (BCA) protein assay kit (Takara, Kusatsu, Shiga, Japan). An equal amount of proteins was separated using 10–12% sodium dodecyl sulfate polyacrylamide gel electrophoresis (SDS-PAGE) and then transferred by electrophoresis to a nitrocellulose membrane (Amersham Biosciences, UK). The membrane was blocked with 5% b non-fat milk for 2 h and incubated with primary antibodies for phosphorylated and total proteins (1:1000 dilution) at 4°C overnight. The antibodies used were the following: rabbit monoclonal anti-p-I*κ*B*α*(#2859S), rabbit polyclonal anti-I*κ*B*α*(#9242S), rabbit polyclonal anti-p-p65(#3031S), rabbit monoclonal anti-p65(#8242S), rabbit monoclonal anti-p-Ikk*β*(#2078S), rabbit monoclonal anti-Ikk*β*(#8943S), rabbit monoclonal anti-NLRP3(#15101S), rabbit monoclonal anti-ASC(#67824S), rabbit polyclonal anti-caspase-1(#2225S), rabbit polyclonal anti-p-PI3K(#4228S), rabbit polyclonal anti-PI3K(#4292S), mouse monoclonal anti-p-AKT(#4051S), mouse monoclonal anti-AKT(#2920S), and rabbit polyclonal anti-*β*-actin(#4967S), and purchased from Cell Signaling Technology (Danvers, MA, USA). After incubation, membranes were washed with TBST three times and incubated with horseradish peroxidase-conjugated secondary antibody (1:2000) for 1 h at room temperature. Blots were washed with TBS-T and an enhanced chemiluminescence kit (Bio-Rad) was used for signal detection. The band intensity was measured using the FluorChem 8000 imaging system (AlphaInnotech, San Leandro, CA, USA).

### 2.6. Statistical Analysis

Statistical analysis was performed using IBM SPSS Statistics 22.0. Results are expressed as mean ± standard error of the mean (SEM) of at least three independent experiments. Statistical significance was evaluated by one-way analysis of variance (ANOVA), Differences were considered statistically significant at a* p* value <0.05.

## 3. Results

### 3.1. Effect of Galangin and UA on NRK-52E Viability

**Figure 1:**
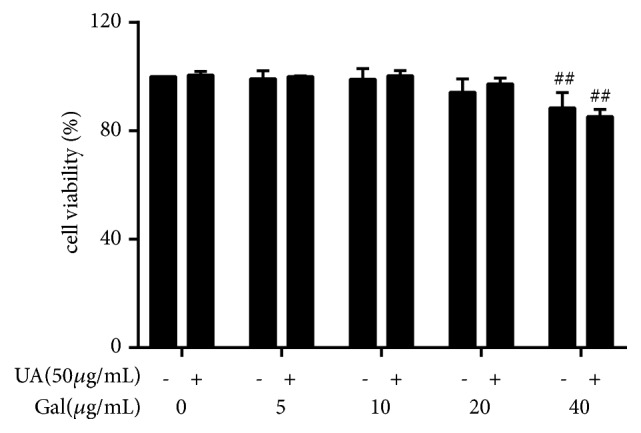
Effect of galangin and UA on NRK-52E viability. NRK-52E cells were treated with galangin (0-40 *μ*g/ml) in the absence or presence of UA for 24 h and cell viability was measured by CCK8 assay. Results are expressed as mean ± SEM of three independent experiments. ##* p*<0.01* vs* control.

The potential cytotoxicity of galangin on NRK-52E cells viability in the absence or presence of UA was evaluated by CCK8 assay. The results showed that galangin did not affect normal cells growth at concentrations up to 20 *μ*g/ml compared to the control group. However, when concentrations were up to 40 *μ*g/ml, galangin displayed cellular toxicity (Figure 1). Thus, for the above reason, concentrations of 5, 10, or 20 *μ*g/ml galangin were chosen for subsequent experiments.

### 3.2. Effect of Galangin on the Release of Pro-Inflammatory Cytokines and Mediators by UA Treated NRK-52E Cells

**Figure 2:**
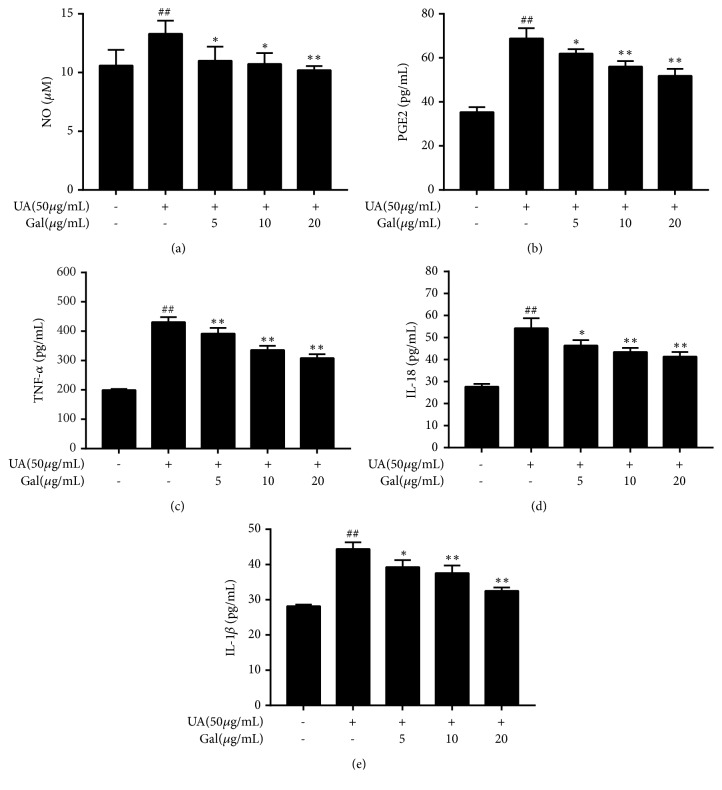
Effect of galangin on the release of pro-inflammatory cytokines and mediators by UA treated NRK-52E cells. NRK-52E cells were treated with UA (50 *μ*g/ml) in the presence of galangin (5, 10, or 20 *μ*g/ml) and untreated cells were considered as the control. The levels of PGE2, TNF-*α*, IL-1*β* and IL-18 were measured by ELISA kit, while NO level was measured by NO kit. Results are expressed as mean ± SEM of three independent experiments. #* p* <0.05, ##* p* <0.01* vs* control; *∗ p* <0.05, *∗∗ p* <0.01* vs* UA group.

To evaluate the anti-inflammatory effect of galangin on UA treated NRK-52E cells, we measured the levels of TNF-*α*, PEG2, IL-1*β*, and IL-18 by ELISA, while the NO level was measured by Griess assay. Compared with the control group, the levels of TNF-*α*, IL-1*β*, IL-18, PGE2, and NO were significantly increased after UA treatment (*p* < 0.01). However, co-treatment with galangin markedly decreased the release of TNF-*α*, IL-1*β*, IL-18, PEG2 and NO. These results showed that galangin inhibited the release of pro-inflammatory cytokines and mediators at all concentrations used in UA treated NRK-52E cells. (*p* < 0.05,* p* < 0.01) (Figure 2).

### 3.3. Effect of Galangin on Pro-Inflammatory Cytokines and Mediators mRNA Expression in UA Treated NRK-52 Cells

**Figure 3:**
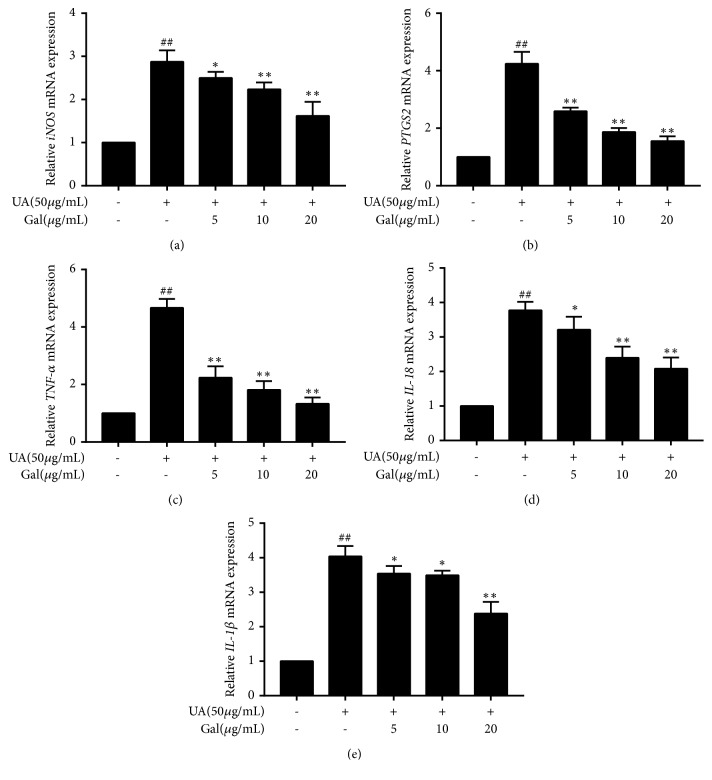
Effect of galangin on pro-inflammatory cytokines and mediators mRNA expression in UA treated NRK-52E cells. NRK-52E cells were treated with UA (50 *μ*g/ml) in the presence of galangin (5, 10, or 20 *μ*g/ml) and untreated cells were considered as the control. Then iNOS, PTGS2, TNF-*α*, IL-1*β* and IL-18 mRNA expression was measured by RT-qPCR. Results are expressed as mean ± SEM of three independent experiments. # p <0.05, ## p <0.01 vs control; *∗* p <0.05, *∗∗* p <0.01 vs UA group.

To investigate whether the effect of galangin on the decreased release of pro-inflammatory cytokines and mediators was associated with the regulation of their mRNAs expression, RT-qPCR was performed on iNOS, PTGS2, TNF-*α*, IL-1*β* and IL-18. The results showed that the mRNA expression of iNOS, PTGS2, TNF-*α*, IL-1*β* and IL-18 was significantly increased in UA treated NRK-52E cells compared to their expression in the control group (*p* <0.01), However, co-treatment with galangin decreased all these mRNAs in UA treated NRK-52E cells, and this effect was statistically significant at all galangin concentrations used compared to the effect on the UA group (*p* < 0.01,* p* < 0.05) (Figure 3).

### 3.4. Effect of Galangin on NF-*κ*B in UA Treated NRK-52E Cells

**Figure 4:**
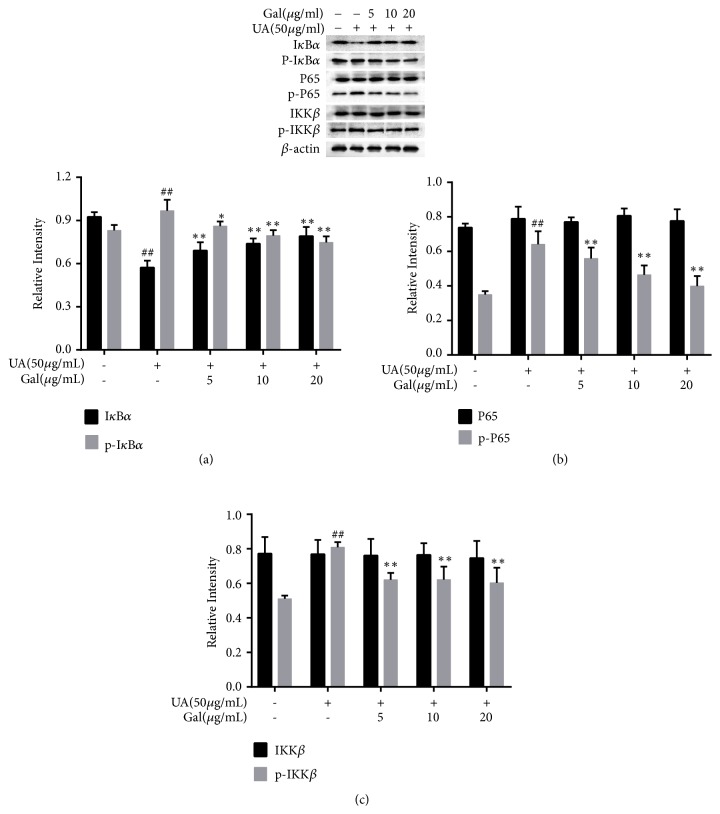
Effect of galangin on NF-*κ*B in UA treated NRK-52E cells. Galangin decreased the expression of the phosphorylated form of I*κ*B*α*, IKK*β*, and p65, and inhibited I*κ*B*α* degradation in UA treated NRK-52E cells. Each of the protein bands was normalized to band of *β*-actin and reported as relative intensity. Results are expressed as mean ± SEM of three independent experiments. #*p* < 0.05, ##*p* < 0.01* vs* control group; *∗p* < 0.05, *∗∗p* < 0.01* vs* UA group.

NF-*κ*B is a crucial regulator in mediating the expression of pro-inflammatory cytokines and mediators. Thus, NF-*κ*B pathway-related protein expression was evaluated by western blotting. As shown in Figure 4, the expression of the phosphorylated form of IKK*β*, I*κ*B*α*, and p65 was markedly upregulated (*p* < 0.01), while I*κ*B*α* expression was markedly downregulated (*p* < 0.01) in UA treated NRK-52E cells, compared to their expression in the control group. Furthermore, Galangin significantly downregulated the expression of the phosphorylated form of these proteins (*p* < 0.01,* p* < 0.05) and upregulated I*κ*B*α* expression compared to the UA group (*p* < 0.01,* p* < 0.05). However, the expression of p65 and IKK*β* was not affected by galangin. These data suggested that galangin might inhibit the activation of NF-*κ*B by reducing the phosphorylated form of p65, I*κ*B*α*, IKK*β*, and by inhibiting the degradation of I*κ*B*α* in UA treated NRK-52E cells.

### 3.5. Effect of Galangin on the Expression of the Phosphorylated Form of PI3K and AKT in UA Treated NRK-52E Cells

**Figure 5:**
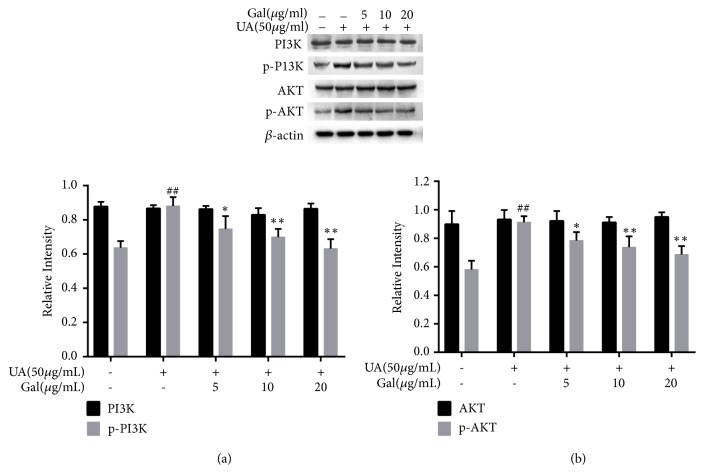
Effect of galangin on the expression of the phosphorylated form of PI3K and AKT in UA treated NRK-52E cells. Galangin decreased the expression of the phosphorylated form of PI3K and AKT in UA treated NRK-52E cells. Each of the protein bands was normalized to band of *β*-actin and reported as relative intensity. Results are expressed as mean ± SEM of three independent experiments. #p < 0.05, ##p < 0.01 vs control group; *∗*p < 0.05, *∗∗*p < 0.01 vs UA group.

The PI3K/AKT pathway also plays an important role in the inflammatory response. To further investigate whether galangin inhibited renal inflammation via the PI3K/AKT, the expression of these proteins was evaluated. As shown in Figure 5, the expression of the phosphorylated form of PI3K and AKT was significantly upregulated by UA compared to the control group (*p* <0.01), and both of them were slightly downregulated following galangin treatment as compared to the UA group (*p* < 0.01,* p* < 0.05). However, both AKT and PI3K were unchanged by UA or galangin treatment. These results suggested that galangin might be involved in PI3K/AKT pathway-mediated inflammatory process in UA treated NRK-52E cells.

### 3.6. Effect of Galangin on NLRP3 Expression in UA Treated NRK-52E Cells

**Figure 6:**
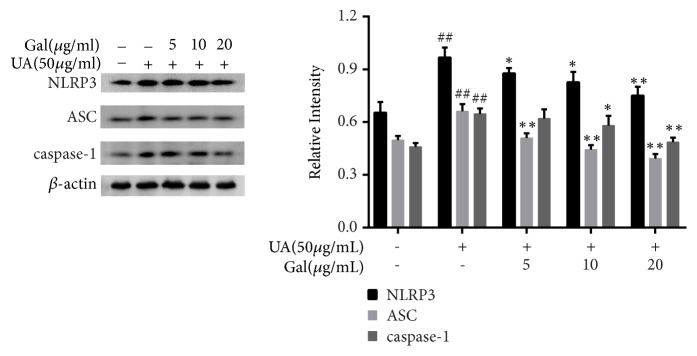
Effect of galangin on NLRP3 expression in UA treated NRK-52E cells. Galangin inhibited the expression of NLRP3, ASC and caspase-1 in UA treated NRK-52E cells. Each of the protein bands was normalized to band of *β*-actin and reported as relative intensity. Results are expressed as mean ± SEM of three independent experiments. #p < 0.05, ##p < 0.01 vs control group; *∗*p < 0.05, *∗∗*p < 0.01 vs UA group.

NLRP3, an inflammasome member, catalyzes the maturation of inflammatory factors such as IL-1*β* and IL-18, Since IL-1*β* and IL-18 were already shown to be reduced by galangin in our research, to further evaluate whether the anti-inflammatory effect of galangin was related with NLRP3 inflammasome in UA treated NRK-52E cells, the expression of related proteins was evaluated. As shown in Figure 6, NLRP3, ASC, and caspase-1 were markedly upregulated compared to their expression in the control group (*p* < 0.01). Meanwhile, galangin significantly downregulated the expression of NLRP3 and ASC compared to their expression in the UA group (p < 0.01, p < 0.05) and downregulated the expression of caspase-1 from a concentration of 10 *μ*g/ml onward compared to its expression in the UA group (*p* < 0.01,* p* < 0.05). These results indicated that galangin may play an anti-inflammatory effect by inhibiting the activation of NLRP3 inflammasome.

## 4. Discussion

*Alpinia officinarum* Hance exerts multiple pharmacological actions including anti-inflammatory, anti-bacterial, anti-fungal, anti-vitiligo effects, in addition to its effect against Alzheimer's disease and cancer. Galangin is the main compound isolated from this plant and has been reported to have anti-inflammatory effects. In this study, we demonstrated its effect against renal inflammation and we clarified its molecular mechanism.

As a main cytotoxic mediator, NO plays a key role in the inflammatory response [24], it is synthesized from L-arginine by iNOS, and both NO and iNOS are expressed at low levels under normal conditions and significantly increase during an inflammatory response[25]. In addition, PGE2, another cytotoxic mediator generated by PTGS2, also mediates the inflammatory response [26]. Many natural products that exhibit inhibitory effect on NO and PGE2 production and suppress iNOS and PTGS2 have been found [27]. Our results demonstrated that galangin reduced UA induced production of NO and PGE2 and downregulated the expression of iNOS and PTGS2 mRNA. Moreover, the stimulated iNOS and PTGS2 can activate pro-inflammatory cytokines such as TNF-*α*, IL-18, and IL-1*β*, which are early pro-inflammatory mediators and modulate inflammatory response [28]. However, overwhelming secretion of pro-inflammatory cytokines causes severe tissue damage, multiple organ failure, or death [29]. Therefore, a therapeutic strategy repressing the overproduction of pro-inflammatory cytokines due to UA is necessary to avoid the complications mentioned above. Our results indicated that galangin reduced TNF-*α*, IL-18, and IL-1*β* mRNA expression induced by UA treatment. Additionally, it has been demonstrated that galangin has almost no cytotoxic activity in V79 cells, a cell line widely used in toxicity studies [30]. Indeed, our results also demonstrated that galangin has anti-inflammatory activity with no significant effect on cell viability below the concentration of 20*μ*g/mL.

NF-*κ*B, a vital transcription factor that has been involved in the inflammatory response[31], not only stimulates pro-inflammatory cytokines such as TNF-*α* and IL-1*β*, but also stimulates pro-inflammatory mediators, such as NO and PGE-2. NF-*κ*B is an early nuclear transcription factor, and p65 is an important subunit of the NF-*κ*B transcription factor family, which plays a central regulatory role in inflammatory response. Under normal physiological conditions, NF-*κ*B exists as an inactive heterodimers composed of p50 and p65 in the cytoplasm by a family of inhibitors, named I*κ*B*α*. When stimulated by other external stimulus, I*κ*B*α* is rapidly phosphorylated by IKK, the upstream kinase for I*κ*B*α*, and subsequently ubiquitinated and degraded. This results in the release of NF-*κ*B from I*κ*B*α*, and transfer to the nucleus, leading to expression of several inflammatory genes such as TNF-*α*, IL-1*β* and NO [32, 33]. As shown in our present study, treating NRK-52E cells with UA leads to the activation of NF-*κ*B, and the activation can be significantly suppressed by galangin through decreasing the expression of the phosphorylated form of I*κ*B*α*, IKK*β*, and p65, as well as inhibiting I*κ*B*α* degradation. These results indicated galangin inhibited UA-induced inflammatory responses likely by inhibiting NF-*κ*B signaling pathway.

In addition, PI3K/Akt pathway is important for the activation of NF-*κ*B as well. PI3K and AKT, the upstream molecules of NF-*κ*B, have been reportedly involved in various cellular processes [34, 35]. AKT is an important target signal molecule in the downstream signaling pathway of PI3K. Studies have shown that AKT can allow nuclear translocation of NF-*κ*B from cytoplasm to nucleus through phosphorylating I*κ*B*α* and activating of the IKK complex, thereby regulating the synthesis and release of inflammatory factors [36]. To better investigate the anti-inflammatory mechanism of galangin, we examined the effect of galangin on PI3K/AKT signaling pathway. Our findings indicate that UA-induced increase in protein levels of phosphorylated form of PI3K and AKT was dramatically decreased by galangin. Taken together, it suggested that galangin plays the anti-inflammatory role through PI3K/Akt/NF-*κ*B signaling pathway.

Recent studies have found that another group of signaling molecules, the NLRP3 inflammasome that includes NLRP3, ASC, and caspase-1, is involved in the production of inflammatory mediators [37, 38]. It is also reported that NF-*κ*B is the important signal I (NF-*κ*B signaling) in NLRP3 inflammasome and regulates its activation [39]. Upon activation, NLRP3 proteins consequently recruits the adapter ASC, which in turn induces the translocation and activation of caspase-1, further resulting in the maturation of inflammatory cytokines IL-1*β* and IL-18 [40]. Recent studies have suggested that the NLRP3 region is implicated in the pathogenesis of more common inflammatory diseases [41], and Shankar et al. discovered that NLRP3 inflammasome activation is involved in the pathogenesis of renal inflammation [42]. To further explore the molecular mechanism, the activity of NLRP3 inflammasome was investigated. As expected, we observed that galangin effectively decreased the levels of IL-1*β* and IL-18 and downregulated NLRP3 protein expression and the inhibition of ASC adaptor as well as caspase-1 activity.

According to our research results, galangin is characterized as a potent candidate, which might attenuate UA-induced inflammation. The conclusions were based on the following: Foremost, galangin can suppress the activation of PI3K/Akt pathway, causing the subsequently inhibitory effect on NF-*κ*B activation. Then, the inhibition of NF-*κ*B activation by galangin can inhibit activation of the NLRP3 inflammasome, which both effectively decreases the levels of pro-inflammatory cytokines, and finally suppresses inflammation.

## 5. Conclusions

In conclusion, the present study demonstrated that galangin might play a key role against renal inflammation by inhibiting inflammatory responses through the inhibition of PI3K/AKT NF-*κ*B, and NLRP3 inflammasome activation. Taken together, our findings provide beneficial evidences in the application of galangin against renal inflammation.

## Data Availability

The data used to support the findings of this study are available from the corresponding author upon request.
